# Decreased Ubiquitination and Acetylation of Histones 3 and 4 Are Associated with Obesity-Induced Disorders of Spermatogenesis in Mice

**DOI:** 10.3390/toxics12040296

**Published:** 2024-04-17

**Authors:** Mahamadou Fofana, Zhenyang Li, Han Li, Wenqi Li, Lu Wu, Lu Lu, Qizhan Liu

**Affiliations:** 1 Center for Global Health, China International Cooperation Center for Environment and Human Health, The Key Laboratory of Modern Toxicology, Ministry of Education, School of Public Health, Nanjing Medical University, Nanjing 211166, China; fchamiko8@gmail.com (M.F.); zhenyang_l@163.com (Z.L.); hanli@stu.njmu.edu.cn (H.L.); njmuliwenqi0311@163.com (W.L.); 2 Suzhou Center for Disease Control and Prevention, Suzhou Institute for Advanced Study of Public Health, Suzhou School, Nanjing Medical University, Suzhou 215004, China; wuluxxl@126.com; 3 Animal Core Facility, The Key Laboratory of Model Animal, Jiangsu Animal Experimental Center for Medical and Pharmaceutical Research, Nanjing Medical University, Nanjing 211166, China

**Keywords:** high-fat diet, spermatogenesis, epigenetic, reproductive disorders

## Abstract

Background: Obesity, a chronic metabolic disorder, is related to cardiovascular diseases, diabetes, cancer, and reproductive disorders. The relationship between obesity and male infertility is now well recognized, but the mechanisms involved are unclear. We aimed to observe the effect of obesity on spermatogenesis and to investigate the role of histone ubiquitination and acetylation modifications in obesity-induced spermatogenesis disorders. Methods: Thirty male C57BL/6J mice were randomly divided into two groups. The control group was fed with a general maintenance diet (12% fat), while a high-fat diet (HFD) group was fed with 40% fat for 10 weeks; then, they were mated with normal females. The fertility of male mice was calculated, testicular and sperm morphology were observed, and the expression levels of key genes and the levels of histone acetylation and ubiquitination modification during spermatogenesis were detected. Results: The number of sperm was decreased, as well as the sperm motility, while the number of sperm with malformations was increased. In the testes, the mRNA and protein expression levels of gonadotropin-regulated testicular RNA helicase (GRTH/DDX25), chromosome region maintenance-1 protein (CRM1), high-mobility group B2 (HMGB2), phosphoglycerate kinase 2 (PGK2), and testicular angiotensin-converting enzyme (tACE) were decreased. Furthermore, obesity led to a decrease in ubiquitinated H2A (ubH2A) and reduced levels of histone H3 acetylation K18 (H3AcK18) and histone H4 acetylation K5, K8, K12, and K16 (H4tetraAck), which disrupted protamine 1 (Prm1) deposition in testis tissue. Conclusion: These results suggest that low levels of histone ubiquitination and acetylation are linked with obesity-induced disorders during spermatogenesis, contributing to a better understanding of obesity-induced damage to male reproduction.

## 1. Introduction

Obesity is a chronic metabolic condition resulting from the interaction of genetic, environmental, and other factors. For obesity, the imbalance between energy intake and energy consumption leads to excessive body weight gain due to abnormal fat accumulation. Obesity elevates the risks of hypertension, diabetes, cardiovascular disease, cancer, and reproductive disorders [1,2]. There has been considerable research in regard to the impact of obesity on female infertility; however, the effect of obesity on male infertility has been rarely explored. The prevalence of obesity and infertility is becoming an increasingly challenging issue for the global health system [3,4]. Recently, clinical and experimental research have revealed effects of obesity on male infertility [5,6].

Obesity is related to inflammation, and a chronic inflammatory state leads to the formation of reactive oxygen species (ROS) in the male reproductive system [7]. At micro-environment levels, obesity has an impact on spermatogenesis in the testis and maturation of sperm in the epididymis. Sertoli and Leydig cells are involved in testicular spermatogenesis. The low levels of testosterone and the inflammation caused by obesity affect the cell junctions between Sertoli cells and cause disorders of the seminal epithelial niche, which is necessary for spermatogenesis [8]. The lipid composition of sperm has an influence on their function, including their motility and viability. For obese males, sperm cholesterol (CHOL) contents are elevated, resulting in abnormal sperm morphology and low sperm motility [9,10]. These factors are associated with decreased sperm quality, including decreased sperm motility and acrosome reaction, elevated ROS and DNA damage, disturbed blood–testis barrier integrity, and aberrant epigenetic modifications, leading to male infertility [1,11].

The mechanism for obesity-induced male infertility is complex and multifaceted. Spermatogenesis is an essential part, for normal spermatogenesis is required for the production of mature sperm. Various molecules are involved in spermatogenesis, including gonadotropin-regulated testicular helicase (*GRTH/DDX25*) and nuclear exporter chromosome region maintenance 1 (*CRM1*). These molecules regulate the expression of high-mobility group B2 (*HMGB2*), phosphoglycerate kinase 2 (*PGK2*), and testicular angiotensin-converting enzyme (*tACE*), which are necessary for spermatid development and the completion of spermatogenesis [12,13]. *DDX25* plays an important role during the process of sperm elongation (in which round spermatids are converted to elongated spermatids) [14]. It is also involved in cholesterol metabolism at the mitochondrial level of male reproductive cells [15]. The process of spermatogenesis in *DDX25* knockout mice is arrested in step 8 when round spermatids fail to elongate [16]. *DDX25* interacts with CRM1 to regulate the nuclear export and post-transcriptional translation of *HMGB2*, *PGK2*, and *tACE* [12].

Epigenetic alterations induced by a Western diet in male germline cells can cause subfertility by affecting sperm quality [12]. Furthermore, offspring can inherit these alterations [17,18,19]. Spermatogenesis also involves the remodeling of chromatin. In this process, most of the histones in spermatozoa are replaced by protamine, leading to the formation of dense chromatin structures that protect the DNA of spermatids from damage [20]. Epigenetic modifications are involved in the process of replacing histones with protamine. The initial step is the ubiquitination of histones H2A and H2B, followed by H4 acetylation and histone removal [21,22]. This ubiquitination–acetylation-to-protamine transition is essential for normal spermatogenesis. However, it is unclear whether histone ubiquitination/acetylation has a role in the reproductive toxicity caused by obesity.

The aim of this study was to investigate the roles of histone acetylation and ubiquitination in obesity-led decline in sperm quality by interfering with the expression of genes involved in spermatogenesis, resulting in a deterioration of fertility for male mice.

## 2. Materials and Methods

### 2.1. Mice and Treatments

For mice, all animal studies and experiments complied with the Guidelines for the Care and Use of Laboratory Animals of the Chinese Animal Welfare Committee and were reviewed and approved by the Institutional Animal Care and Use Committee of Nanjing Medical University (approval number: IACUC-2211004). Thirty male C57BL/6J mice (8 weeks old) were obtained from the Animal Core Facility of Nanjing Medical University and housed in the animal facilities of the Safety Assessment and Research Center for Drug, Pesticide and Veterinary Drug of Jiangsu Province. After acclimatization for one week, mice were randomly divided into a control group and an HFD group. The control group was fed with a general maintenance diet (12% fat); the high-fat group was fed with an HFD (40% fat). For 10 weeks, the mice were free to eat and drink sterile water. The mice were kept in a temperature-controlled environment and subjected to a 12 h light/dark cycle (lights on at 8 a.m.). Each week, the mice were weighed, and their food and water intakes were recorded. After the establishment of male obese mice, these mice were mated with 6-week-old normal females (1 male against 2 females). Female mice with copulatory plugs were considered as pregnant. The fertility of the obese male mice was calculated as follows: (number of delivering mice)/(number of pregnant mice), and the births of their offspring were recorded.

### 2.2. Body Composition Analysis

Based on previous studies [23,24], the body composition of mice was analyzed by time-domain nuclear magnetic resonance (TD-NMR). This non-invasive method involved the use of a BRUKER Minispec LF50 (mq7.5) instrument (BRUKER, Billerica, MA, USA). The mice, without anesthesia, were weighed and placed into a special tube of the instrument, adjusting the piston of the tube to restrict their movement. The tube was then placed into the probe of the BRUKER instrument, and, for the mouse, the body fat mass, lean mass, and free body fluid were determined. Each mouse was measured 3 times. The measurements were not taken continuously; before each measurement, the sample tube was removed from the instrument, so that excessive sweating and/or dehydration induced by the high temperature inside the probe were avoided. The following calculations were made: adiposity index (%) = body fat mass (g)/body weight (g) × 100%; lean body mass index (%) = lean body mass (g)/body weight (g) × 100%; lean/fat ratio = lean body mass (g)/body fat mass (g).

### 2.3. Measurement of Serum Lipid Levels

After mice were fed with an HFD for 10 consecutive weeks, blood was collected from the inner canthus, which was allowed to remain at room conditions for 1 to 2 h, then centrifuged at 4 °C for 30 min at 3000 rotations per min. The resulting supernatant was collected as serum and stored at −80 °C. To evaluate lipids, we measured the levels of free fatty acids, triglycerides, low-density lipoprotein cholesterol (LDL-C), high-density lipoprotein cholesterol (HDL-C), and total cholesterol (TC) using the corresponding assay kits (Nanjing Jiancheng, Nanjing, China), following the reagent kit instructions.

### 2.4. Testis Index and Histology

Mice from each group were randomly selected, and their bilateral testes were removed after cervical dislocation. The weight and size of the testes were measured, and the testis index was determined as (testis weight/body weight) × 100%. The mice testes were fixed using 4% paraformaldehyde and embedded in paraffin. Sections of 5 µm were placed on slides and stained with hematoxylin and eosin (H&E) and periodic acid–Schiff (PAS), following the manufacturer’s instructions. From each group, 6 slices and 10 fields were randomly selected for microscopic assessment of histological changes. 

### 2.5. Immunohistochemistry (IHC) Assay

The testes were sectioned into 3 μm-thick slices and fixed in paraffin. After deparaffinization and rehydration, the sections were prepared for immunohistochemistry (IHC) staining using primary antibodies against either ubiquitinated H2A (ubH2A) or protamine 1 (Prm1) following a blocking and antigen retrieval procedure. The histopathology slides were scanned at a magnification of 40× using Panoramic Scan (3DHISTECH Ltd., Budapest, Hungary). The staining intensity was scored on a scale of 0 to 3, where 0 indicates no staining, 1 indicates weak staining (light yellow), 2 indicates moderate staining (yellow–brown), and 3 indicates strong staining (brown). The percentage of positively stained cells was scored as follows: 1 (<10%), 2 (10–50%), 3 (50–75%), or 4 (>75%). The staining index was calculated by multiplying the staining intensity score by the percentage of positive cells. The index value ranges from 0 to 12.

### 2.6. Sperm Count, Motility, and Malformation

For each mouse, the head of the epididymis was removed and placed in PBS at 37 °C. The epididymal head was pierced to free the mature sperm. Portions of the sperm suspension were placed in a CASA analyzer (Hamilton-Thorne Research, Inc., Beverly, MA, USA). The percentages of motile sperm were determined by the following: (number of motile sperm)/(number of total sperm) × 100%. Further, portions of the sperm suspension (10 μL) were placed onto slides, spread with a cover glass, and allowed to dry. After the preparations were fixed with methanol for 10 min and stained with eosin for 1 h, the relative numbers of malformed sperm were recorded with a Pannoramic Scan (3DHISTECH Ltd., Budapest, Hungary). The ratio of sperm malformations was calculated as (number of abnormal sperm)/(number of total sperm) × 100%.

### 2.7. Transmission Electron Microscopy (TEM)

Sperm suspensions were centrifuged (1500 R/min, 5 min), and the supernatant was discarded. This procedure was triplicated. For the samples, 1–2 mL of 3.1% glutaraldehyde was added, and the preparations were refrigerated at 4 °C. After 2 h of fixation, the preparations were transferred to sample bottles, washed three times with 0.067 M buffer solution, and placed at 4 °C overnight. The sperm samples were then fixed, dehydrated, and embedded in EPON 812 at 60 °C for 24 h. In addition, ultrathin sections were double-stained with uranium and lead [25]. The acrosome morphology of sperm was evaluated with an electron microscope (JEM-1200EX, JEOL Ltd., Tokyo, Japan). 

### 2.8. Reverse Transcription PCR (RT-PCR) 

Total RNA from testes of male mice was isolated by use of Trizol reagent (Thermo Fisher Scientific, Waltham, MA, USA), according to the manufacturer’s protocol. The A260/280 ratio was in the range of 1.8–2.0, and the concentration was measured with NanoDrop 2000 (Thermo Fisher, Waltham, MA, USA). A total of 1 μg of total RNA extract and HiScript II Q RT Supermix (Vazyme Biotech, Nanjing, China) were used in reverse transcription; the primers are listed in Table 1. To agarose gels (1.2%), 5 µL of GelRed Nucleic Acid Stain (Yeasen Biotechnology Co., Ltd., Shanghai, China) was added, and samples were subjected to electrophoresis for 20 min at 200 V, 100 mA. The fluorescent ethidium bromide-stained DNA separation pattern was imaged using a Molecular Imager ChemiDoc XRS System (Bio-Rad, Hercules, CA, USA).

### 2.9. Western Blots

Total protein was extracted from mice testis tissues using RIPA lysis buffer (Beyotime Institute of Biotechnology, Shanghai, China). BCA kits (Beyotime Institute of Biotechnology in Shanghai, China) were used to determine protein concentrations (Beyotime Institute of Biotechnology, Shanghai, China). Subsequently, 30 μg of protein was loaded into 10% SDS-PAGE, then electrotransferred to PVDF membranes (Millipore, Burlington, MA, USA). The membranes were incubated overnight at 4 °C with a 1:1000 dilution of mouse anti-glyceraldehyde 3-phosphate dehydrogenase (anti-GAPDH, Sigma, St. Louis, MO, USA) and an antibody for GRTH/DDX25 (Santa Cruz Biotechnology, Shanghai, China), PGK2 (Abcam, Washington, DC, USA), HMGB2, CRM1 (Abcam, USA), Prm1 (Abcam, USA), tACE (Beyotime Institute of Biotechnology, Shanghai, China), H3 (Abcam, USA), H4 (Abcam, Washington, DC, USA), H2A (Abcam, USA), ubH2A (Abcam, Washington, DC, USA), H3AcK18 (Abcam, Washington, DC, USA), or H4tetraAcK (Abcam, Washington, DC, USA). Following additional incubation with a 1:1000 dilution of a horseradish peroxidase-linked anti-immunoglobin antibody for 1 h, the immune complexes were detected using enhanced chemiluminescence (Cell Signaling Technology, Boston, MA, USA). The protein bands on the blots were measured for densitometric analyses using Image J software (v1.6, National Institutes of Health, Washington, MA, USA) for densitometric analyses.

### 2.10. Statistical Analysis

The results were presented as mean ± SD. Student’s *t*-tests were used for comparing two groups, and one-way analysis of variance (ANOVA) followed by least significant difference (LSD) was used for multi-group comparisons. A statistically significant result was defined as having a *p*-value of less than 0.05. The statistical tests were performed using GraphPad Prism 7 software (GraphPad Software Inc., San Diego, CA, USA). 

## 3. Results

### 3.1. Establishment of a Model for Obese Male Mice

**Figure 1:**
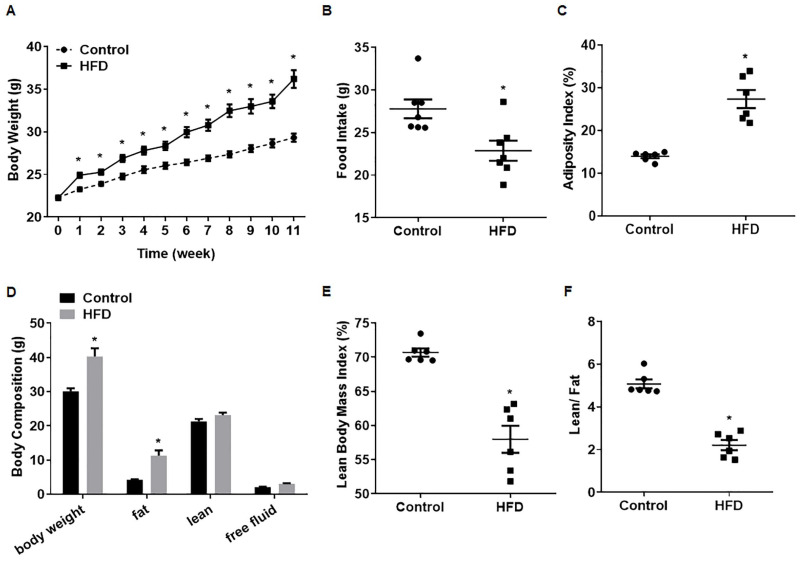
Establishment of a model for obese male mice. Male mice were fed with a normal diet (12% fat) or an HFD (40% fat) for 10 weeks; body weights and food intake were recorded weekly. (**A**) Body weights of male mice (n = 15). (**B**) Food intake of mice, n = 7; the results are presented as mean ± SD, * *p* < 0.05 compared to the control. For mice, adiposity (**C**), body composition (**D**), lean body mass indices (**E**), and lean/fat ratios (**F**) were calculated, n = 6, mean ± SD. * *p* < 0.05 compared to the control.

After 1 week, the body weights of mice on an HFD were elevated compared with those of control mice, and this effect maintained until the end of the experiment (Figure 1A). The food intake tended to be lower in the HFD group, perhaps due to the greater energy provided by the HFD per unit of mass (Figure 1B). Measures of the body composition of mice showed that the adiposity index (Figure 1C) and the absolute fat mass of HFD mice (Figure 1D) were higher. Despite a slightly higher absolute lean weight, the lean body mass index and the lean/fat ratio were lower (Figure 1E,F), showing that the weight gain for HFD mice was mainly due to a higher fat mass. Thus, for male mice, a model of HFD-induced obesity was developed.

### 3.2. The Serum Lipid Contents Were Altered in Mice with HFD-Induced Obesity

**Figure 2:**
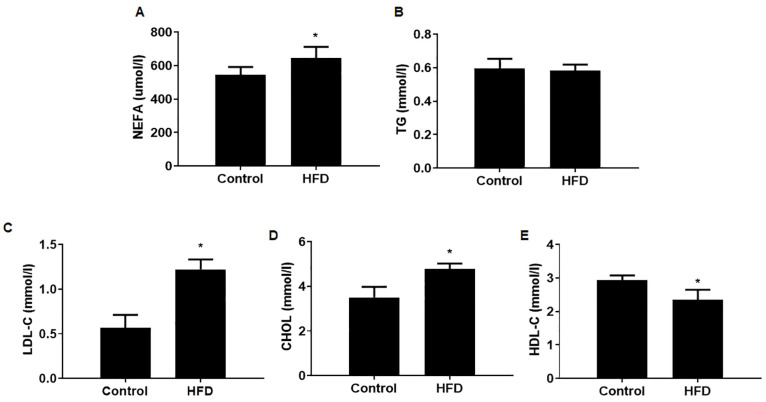
Serum lipid contents were altered in mice with HFD-induced obesity. Male mice were fed with a control diet (12% fat) or high-fat diet (40% fat) for 10 weeks and the serum lipid contents determined. The concentrations of NEFA (**A**), TG (**B**), LDL-C (**C**), CHOL (**D**), and HDL-C (**E**) were determined, n = 6, mean ± SD, * *p* < 0.05 compared to the control. Abbreviations: NEFA, nonesterified fatty acids; TG, triglyceride; LDL-C, low-density lipoprotein; CHOL, cholesterol; HDL-C, high-density lipoprotein.

To investigate whether the observed differences in body weight gain of male mice are reflected in lipid serum composition, mice were sacrificed after 10 weeks of diet and the serum lipid contents of male mice determined. For obese mice, the serum levels of nonesterified fatty acids (NEFAs), LDL-C, and CHOL were substantially higher; the levels of serum HDL-C were moderately lower; and TG levels were unchanged (Figure 2). These results validate the establishment, for male mice, of a model for obesity induced by an HFD.

### 3.3. The Fertility Was Decreased in Mice with HFD-Induced Obesity

To investigate the impact of obesity on male mice reproduction function, the mice with HFD-induced obesity were mated with normal females (6 weeks old). We assessed the fertility of the male mice and recorded the births of their offspring. The fertility of HFD male mice was decreased compared to the control group (Table 2). Further, the litter weights and the litter sizes of newborn offspring were lower in the HFD group. These results show that obesity induced by an HFD reduces fertility in male mice.

### 3.4. The Testes Were Damaged in Mice with HFD-Induced Obesity

**Figure 3:**
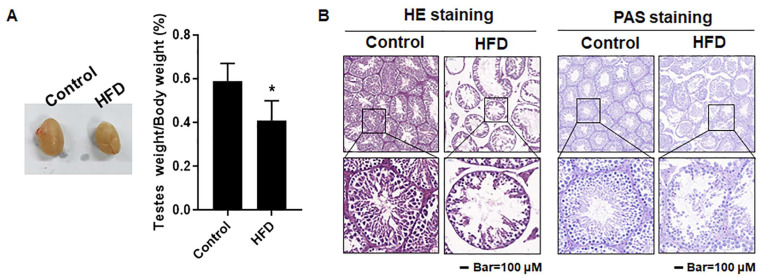
The testes were damaged in mice with HFD-induced obesity. Male mice were fed with a normal diet (12% fat) or an HFD (40% fat) for 10 weeks, and testes were taken after sacrifice. (**A**) The size of testes and the testis index, expressed as (testis weight/body weight) × 100%, n = 6; results are presented as mean ± SD, * *p* < 0.05 compared to the control. (**B**) Testicular histology was determined by H&E and PAS staining. Scale bar = 100 μm (Upper) and 20 μm (Lower).

Abnormal testicular histology is a characteristic of infertility [26]. To evaluate the impact of obesity on spermatogenesis, the testes were dissected and the ultrastructure was observed. We found that the testes size and the testis index of HFD mice were lower (Figure 3A), the gaps among seminiferous tubules testis were higher, and the arrangement of spermatids was disorganized (Figure 3B). These results established that, for male mice, the obesity induced by an HFD caused testicular damage, affecting tissues associated with spermatogenesis.

### 3.5. The Sperm Quality Was Lower in Mice with HFD-Induced Obesity

**Figure 4:**
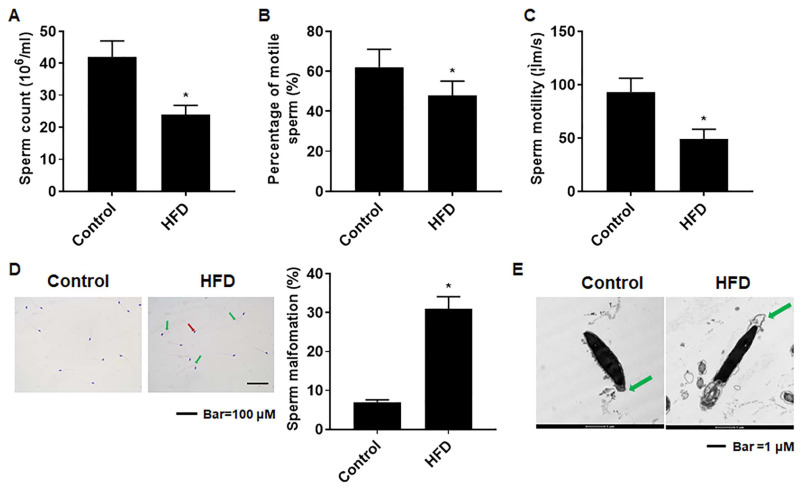
Sperm quality was lower and sperm malformations were elevated in mice with HFD-induced obesity. Male mice were fed with a normal diet (12% fat) or an HFD (40% fat) for 10 weeks. After the sacrifice of the mice, the epididymides were removed. (**A**) Total sperm counts, (**B**) percentages of motile sperm (%), as well as (**C**) sperm motility (μm/s) were assessed, n = 6; all results are presented as mean ± SD, * *p* < 0.05 compared to the control. (**D**) Sperm morphology as determined by staining with H&E (Scale bar = 100 μm) and the corresponding ratios of sperm malformations in the various groups were determined, mean ± SD, * *p* < 0.05 compared to the control. (**E**) TEM images of sperm; the green arrow shows a defective acrosome. Scale bar = 1 μm.

In the study of mature sperm from the epididymis, we found that sperm counts were lower for the HFD group (Figure 4A); both the percentage of motile sperm and sperm motility were also lower (Figure 4B,C). Further, sperm malformations were evident in obese mice. Some showed the sperm head folded back, pointing to the tail tip, and some sperm heads lacked a hook. Further, for obese mice, the sperm malformation rate was elevated (Figure 4D). In addition, analysis of sperm ultrastructure revealed that, for obese mice, the acrosomal integrity of sperm was affected (Figure 4E). These results demonstrated that, for male mice, obesity induced by an HFD disordered spermatogenesis, resulting in reduced sperm quality and an elevated occurrence of sperm malformations.

### 3.6. The mRNA and Protein Levels of Genes Associated with Spermatogenesis Were Decreased in Testes of Obese Mice

**Figure 5:**
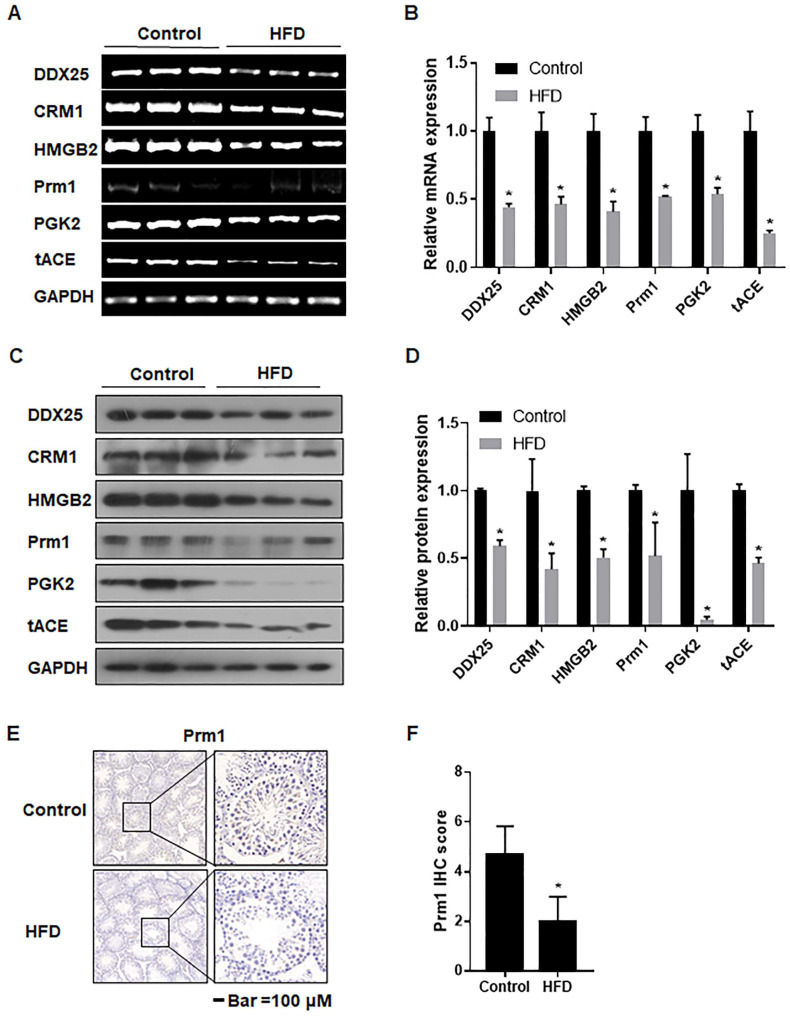
The mRNA and protein levels of genes associated with spermatogenesis were decreased in testes of obese mice. Male mice were fed with a normal diet (12% fat) or an HFD (40% fat) for 10 weeks. (**A**) RT-PCR was performed, and (**B**) relative mRNA expressions levels of DDX25, CRM1, HMGB2, Prm1, PGK2, and tACE in testes were determined, n = 6, mean ± SD, * *p* < 0.05 compared to the control. (**C**) Western blots were performed, and (**D**) protein expression levels of DDX25, CRM1, HMGB2, Prm1, and PGK2 in testes of the indicated group were determined, n = 3; values are presented as mean ± SD, * *p* < 0.05 compared to the control. (**E**) IHC staining of Prm1 in the testes of mice. Scale bar = 100 μm (Left) and 20 μm (Right). (**F**) The levels of Prm1 were determined by immunoreactive scoring (IRS) of IHC analyses, n = 6; values are for mean ± SD, * *p* < 0.05 compared to the control.

To determine the process in spermatogenesis affected by obesity, we measured the expression of genes associated with spermatogenesis. The mRNA levels of *DDX25*, *CRM1*, and their related genes *HMGB2*, *Prm1*, *PGK2*, and *tACE* in the testes of mice of the HFD group were diminished (Figure 5A,B). A decreased expression level of these proteins was also noted (Figure 5C,D). Further, the deposition of *Prm1* was less extensive (Figure 5E,F). These results indicated that obesity induced by an HFD affected spermatogenesis, in particular, the process of sperm elongation.

### 3.7. The Ubiquitination and Acetylation of Histones 3 and 4 in Testes Were Diminished in Male Mice with HFD-Induced Obesity

**Figure 6:**
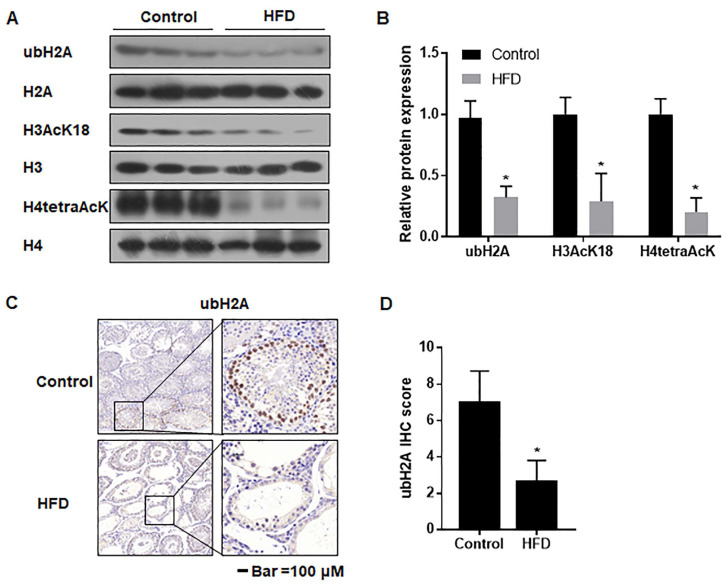
The ubiquitination and acetylation of histones 3 and 4 in testes of male mice with HFD-induced obesity. Mice were fed with a normal diet (12% fat) or an HFD (40% fat) for 10 weeks. (**A**) Western blots were performed, and (**B**) relative protein expression levels of ubH2A, H3AcK18, and H4tetreAcK were evaluated, n = 3; values are for mean ± SD, * *p* < 0.05 compared to the control. (**C**) IHC staining of ubH2A in the testes of mice. Scale bar = 100 μm (Left) and 20 μm (Right). (**D**) The levels of ubH2A as shown by IHC analyses, n = 3; data are expressed as the means ± SD, * *p* < 0.05 compared to the control.

In order to examine the impact of epigenetic alteration during spermatogenesis especially, we measured the acetylation levels of histones H3 and H4 and ubiquitination of H2 in the testes of obese male mice by using immunoblot and RT-PCR. These results indicated that the protein levels of ubH2A, H3AcK18, and H4tetraAcK were lower than those of controls (Figure 6A,B). IHC also revealed lower levels of ubH2A in the testes of HFD mice (Figure 6C,D). These findings showed that obesity induced by an HFD interfered with spermatogenesis by affecting histone modifications.

## 4. Discussion

The impact of obesity, a chronic metabolic disease of global significance, on the reproductive system has been confirmed in clinical and animal experiments. Research is gradually shifting from the effects of obesity on the female reproductive system to the effects on males [2,27]. Obesity causes testicular damage and a decline in sperm quality [2,28], but the influence of obesity on molecules associated with histone modifications in the process of spermatogenesis is not clear. Therefore, we established a mouse model of obesity induced by an HFD, focusing on the effect of obesity on spermatogenesis in male mice, particularly on associated changes in related sperm enzymes and sperm morphology.

In our study, male C57BL/6J mice fed with a Western diet (40% fat) exhibited sustained body weight gain over 10 weeks but reduced food intake, probably due to the high calories provided by an HFD. This contrasts with other studies that have reported weight differences after 12-to-24 weeks of high-fat feeding [29,30]. The genetic, stress-induced reactions; water intake; age; environmental factors; mouse density; and high-calorie content of food lipids, which provide more calories per gram than carbohydrates and proteins, have been found to influence body weight and body composition during experimentation [31,32]. Obesity is characterized by lipid disturbances, such as high levels of TG and TC, low levels of HDL-C, and an abnormal LDL-C composition, associated with excessive weight gain [33,34]. We also observed abnormal serum lipid levels in male mice fed with an HFD.

After construction of a mouse model of obese male mice, we found, by assessing the birth of their offspring, that the fertility of obese male mice was lower than normal male mice; that the testes of obese male mice were smaller than those of normal mice; that the gaps between seminiferous tubules were enlarged; and that the arrangement of spermatogenic cells was disordered. Further, the number of mature sperm in the epididymis was decreased, sperm motility was diminished, and there were more sperm malformations. By use of TEM, we found that the acrosome structure of the spermatozoa of obese male mice was damaged. Gómez-Elías et al. [35] and Jia et al. [36] have reported similar results, suggesting that obesity reduces the fertility of male mice by reducing the quality of mature sperm.

Spermatogenesis is a complex process that involves morphological changes, including the transition of spermatogonia to spermatocytes, to round spermatids, and finally, to mature spermatids [37,38]. The final morphological change is considered to be sperm elongation, the 8th step in spermatogenesis. DDX25-null mice are sterile since spermatogenesis is arrested at the 8th step, which means that *DDX25* regulates sperm elongation [39]. It is also involved in cholesterol metabolism at the mitochondrial level of male reproductive cells [15]. *GRTH/DDX25*, a testis-specific member of the DEAD (Asp–Glu–Gly–Asp)-box family of RNA helicases, is regulated by gonadotropins and is generally present in Leydig and germ cells [40]. It participates in the metabolic processing of RNA, including its transcription, transport, and translation [41,42]. The expression of each gene in spermatogenesis is tightly regulated; two-thirds of mRNAs in the testis of adult mammals combine with its targeted proteins to form messenger ribonucleoproteins (mRNPs), which are released and translated at specific times [43]. In germ cells, *DDX25* is an integral component of mRNPs. It binds to specific mRNAs (those for *HMGB2*, *PGK2*, *Prm1*, and *tACE*), and together with *CRM1*, they regulate the nuclear export and expression of these mRNAs at various stages of spermatogenesis [40,44].

*CRM1*, a nuclear transport protein that moves proteins and RNA from the nucleus, is a component of mRNPs [40,45]. *HMGB2*, a protein that bends linear DNA, is involved in the assembly of nuclear protein complexes and in DNA repair [46]. *PGK2*, a germ cell-specific protein, is translated and expressed during the haploid stage of spermatogenesis, providing a phosphoglycerate kinase essential for the motility and fertility of mammalian spermatozoa [47,48]. *tACE*, a testis-specific angiotensin-converting enzyme, exists only in haploid spermatocytes after meiosis and is expressed during sperm capacitation [49]. The present results showed that, for testes of obese mice, the f mRNA levels and protein expressions of *DDX25*, *CRM1*, and their related genes *HMGB2*, *PGK2*, and *tACE* were low. They also revealed that obesity affects the expression of key genes during the haploid phase of spermatogenesis and that it arrests the elongation of sperm.

The regulation of gene expression through epigenetic mechanisms is essential for normal spermatogenesis [50,51]. During spermatogenesis, a key step is the remodeling of sperm chromatin; round sperm cells undergo nuclear agglutination. During this process, transition proteins, TNP1 and TNP2, replace acetylated histones, which are then replaced by protamine in the sperm cells; the resulting dense structure protects the DNA of sperm cells [52]. Furthermore, the initial step is the ubiquitination of histones H2A and H2B, followed by H4 acetylation and histone removal [21,52,53,54]. During spermatogenesis, there is a high degree of histone acetylation and ubiquitination that opens DNA double strands, facilitating the replacement of histones by protamine. This process is crucial for the compaction of DNA into the sperm head [55]. As revealed in the present study, histone acetylation and ubiquitination, as well as prm1 in sperm cells of obese mice, were lower than that in the control group. These suggest that obesity in male mice inhibits the replacement of histones by protamine, making sperm DNA more vulnerable to damage from external factors [56,57]. Consequently, these lead to a decrease in sperm quality and fertility.

## 5. Conclusions

In conclusion, the present results show that, during spermatogenesis, obesity affects the expression of key genes, arrests the process of sperm elongation, and interferes with normal histone modifications. This process is manifested in low numbers and quality of sperm, resulting in a decline in the fertility of male mice. This appears to be a mechanism by which obesity causes male infertility. The results of our investigation point to the value of further investigations of obesity-induced damage to male reproduction.

## Figures and Tables

**Table 1:** Primer sequences used.

Gene of Interest	Primer Sequence
*DDX25*	*F: 5′-ATGGCGTCGTTACTTTGGGG-3′*
	*R: 5′-AGAGCCGTCTATGTTTGGGAC-3′*
*CRM1*	*F: 5′-CTGCTTGATTTCAGCCAAAAACT-3′*
	*R: 5′-GTATTTCGTGTTCATGTTCTGCG-3′*
*HMGB2*	*F: 5′-AAGAGCGACAAAGCTCGTTATG-3′*
	*R: 5′-GCAGTATCTCCAATAGACAGGC-3′*
*PGK2*	*F: 5′-CCACCTCCAATGGCTGTATC-3′* *R: 5′-CTACCCCTGGAAGGGTTTTG-3′*
*tACE*	*F: 5′-AGTATGACCGGACAGCCAAG-3′* *R: 5′-CCAGGTGCCATATTTCAAGG-3′*
*Prm1*	*F: 5′- CCGTCGCAGACGAAGATGTC-3′* *R: 5′-CACCTTATGGTGTATGAGCGG-3′*
*GAPDH*	*F: 5′-GTCTTCACTACCATGGAGAAGG-3′* *R: 5′-TCATGGATGACCTTGGCCAG-3′*

**Table 2:** Fertility of mice with HFD-induced obesity was decreased.

	Control	HFD
Fertility (%)	100 (30/30)	73.33 (22/30) *
Litter size (n = 15)	8.56 ± 0.72	6.54 ± 1.21 *
Litter weight (g) (n = 15)	9.14 ± 1.38	8.01 ± 1.14 *

Female mice with copulatory plugs were considered pregnant mice, and fertility was calculated as (number of delivering mice)/(number of pregnant mice). Values are expressed as percentages (%). Litter weights and litter sizes of offspring were determined. Values are for means and SD. * *p* < 0.05 compared to the control.

## Data Availability

Data are available upon request.

## References

[1] Liu Y., Ding Z. (2017). Obesity, a serious etiologic factor for male subfertility in modern society. Reproduction.

[2] Deshpande S.S., Nemani H., Pothani S., Khambata K., Kumar A., Kallamadi P.R., Balasinor N.H. (2019). Genetically Inherited Obesity and High-Fat Diet-Induced Obesity Differentially Alter Spermatogenesis in Adult Male Rats. Endocrinology.

[3] Dieases G.B. (2020). High body-mass index—Level 2 risk. Lancet.

[4] Phelps N.H., Singleton R.K., Zhou B., Heap R.A., Mishra A., Bennett J.E., Paciorek C.J., Lhoste V.P.F., Carrillo-Larco R.M., Stevens G.A. (2024). Worldwide trends in underweight and obesity from 1990 to 2022: A pooled analysis of 3663 population-representative studies with 222 million children, adolescents, and adults. Lancet.

[5] Davidson L.M., Millar K., Jones C., Fatum M., Coward K. (2015). Deleterious effects of obesity upon the hormonal and molecular mechanisms controlling spermatogenesis and male fertility. Hum. Fertil..

[6] Venigalla G., Ila V., Dornbush J., Bernstein A., Loloi J., Pozzi E., Miller D., Ramasamy R. (2023). Male obesity: Associated effects on fertility and the outcomes of offspring. Andrology.

[7] Henkel R.R. (2011). Leukocytes and oxidative stress: Dilemma for sperm function and male fertility. Asian J. Androl..

[8] Zhang H., Yin Y., Wang G., Liu Z., Liu L., Sun F. (2014). Interleukin-6 disrupts blood-testis barrier through inhibiting protein degradation or activating phosphorylated ERK in Sertoli cells. Sci. Rep..

[9] Schisterman E.F., Mumford S.L., Chen Z., Browne R.W., Boyd Barr D., Kim S., Buck Louis G.M. (2014). Lipid concentrations and semen quality: The LIFE study. Andrology.

[10] Martínez-Soto J.C., Landeras J., Gadea J. (2013). Spermatozoa and seminal plasma fatty acids as predictors of cryopreservation success. Andrology.

[11] Wang S., Qian Z., Ge X., Li C., Xue M., Liang K., Ma R., Ouyang L., Zheng L., Jing J. (2022). LncRNA Tug1 maintains blood-testis barrier integrity by modulating Ccl2 expression in high-fat diet mice. Cell. Mol. Life Sci..

[12] Han Y.L., Liang C., Manthari R.K., Yu Y.X., Gao Y., Liu Y., Jiang S.S., Tikka C., Wang J.D., Zhang J.H. (2020). Arsenic influences spermatogenesis by disorganizing the elongation of spermatids in adult male mice. Chemosphere.

[13] Tsai-Morris C.-H., Koh E., Sheng Y., Maeda Y., Gutti R., Namiki M., Dufau M.L. (2007). Polymorphism of the GRTH/DDX25 gene in normal and infertile Japanese men: A missense mutation associated with loss of GRTH phosphorylation. Mol. Hum. Reprod..

[14] Kavarthapu R., Dufau M.L. (2015). Germ Cell Nuclear Factor (GCNF/RTR) Regulates Transcription of Gonadotropin-Regulated Testicular RNA Helicase (GRTH/DDX25) in Testicular Germ Cells--The Androgen Connection. Mol. Endocrinol..

[15] Fukushima M., Villar J., Tsai-Morris C.-H., Dufau M.L. (2011). Gonadotropin-regulated testicular RNA helicase (GRTH/DDX25), a negative regulator of luteinizing/chorionic gonadotropin hormone-induced steroidogenesis in Leydig cells: Central role of steroidogenic acute regulatory protein (StAR). J. Biol. Chem..

[16] Raju M., Hassan S.A., Kavarthapu R., Anbazhagan R., Dufau M.L. (2019). Characterization of the Phosphorylation Site of GRTH/DDX25 and Protein Kinase A Binding Interface Provides Structural Basis for the Design of a Non-Hormonal Male Contraceptive. Sci. Rep..

[17] Wang T., Gao H., Li W., Liu C. (2019). Essential Role of Histone Replacement and Modifications in Male Fertility. Front. Genet..

[18] Karahan G., Martel J., Rahimi S., Farag M., Matias F., MacFarlane A.J., Chan D., Trasler J. (2023). Higher incidence of embryonic defects in mouse offspring conceived with assisted reproduction from fathers with sperm epimutations. Hum. Mol. Genet..

[19] Deshpande S.S.S., Bera P., Khambata K., Balasinor N.H. (2023). Paternal obesity induces epigenetic aberrations and gene expression changes in placenta and fetus. Mol. Reprod. Dev..

[20] Fullston T., Ohlsson-Teague E.M., Print C.G., Sandeman L.Y., Lane M. (2016). Sperm microRNA Content Is Altered in a Mouse Model of Male Obesity, but the Same Suite of microRNAs Are Not Altered in Offspring’s Sperm. PLoS ONE.

[21] Hao S.L., Ni F.D., Yang W.X. (2019). The dynamics and regulation of chromatin remodeling during spermiogenesis. Gene.

[22] Lu L.-Y., Wu J., Ye L., Gavrilina G.B., Saunders T.L., Yu X. (2010). RNF8-Dependent Histone Modifications Regulate Nucleosome Removal during Spermatogenesis. Dev. Cell.

[23] Gou L.-T., Kang J.-Y., Dai P., Wang X., Li F., Zhao S., Zhang M., Hua M.-M., Lu Y., Zhu Y. (2017). Ubiquitination-Deficient Mutations in Human Piwi Cause Male Infertility by Impairing Histone-to-Protamine Exchange during Spermiogenesis. Cell.

[24] Gordon C.J., Phillips P.M., Johnstone A.F.M. (2016). A noninvasive method to study regulation of extracellular fluid volume in rats using nuclear magnetic resonance. Am. J. Physiol. Renal Physiol..

[25] Li M., Vassiliou C.C., Colucci L.A., Cima M.J. (2015). (1)H nuclear magnetic resonance (NMR) as a tool to measure dehydration in mice. NMR Biomed..

[26] Han Y., Yu Y., Liang C., Shi Y., Zhu Y., Zheng H., Wang J., Zhang J. (2019). Fluoride-induced unrestored arrest during haploid period of spermatogenesis via the regulation of DDX25 in rats. Environ. Pollut..

[27] Su L., Patti M.E. (2019). Paternal Nongenetic Intergenerational Transmission of Metabolic Disease Risk. Curr. Diabetes Rep..

[28] Oliveira P.F., Sousa M., Silva B.M., Monteiro M.P., Alves M.G. (2017). Obesity, energy balance and spermatogenesis. Reproduction.

[29] Zhang X.Y., Guo C.C., Yu Y.X., Xie L., Chang C.Q. (2020). Establishment of high-fat diet-induced obesity and insulin resistance model in rats. Beijing Da Xue Xue Bao Yi Xue Ban.

[30] Komninos D., Ramos L., van der Heijden G.W., Morrison M.C., Kleemann R., van Herwaarden A.E., Kiliaan A.J., Arnoldussen I.A.C. (2022). High fat diet-induced obesity prolongs critical stages of the spermatogenic cycle in a Ldlr−/−.Leiden mouse model. Sci. Rep..

[31] Wang C.Y., Liao J.K. (2012). A mouse model of diet-induced obesity and insulin resistance. Methods Mol. Biol..

[32] Messa G.A.M., Piasecki M., Hurst J., Hill C., Tallis J., Degens H. (2020). The impact of a high-fat diet in mice is dependent on duration and age, and differs between muscles. J. Exp. Biol..

[33] Xu P., Ying L., Hong G., Wang Y. (2016). The effects of the aqueous extract and residue of Matcha on the antioxidant status and lipid and glucose levels in mice fed a high-fat diet. Food Funct..

[34] Franssen R., Monajemi H., Stroes E.S.G., Kastelein J.J.P. (2011). Obesity and dyslipidemia. Med. Clin. N. Am..

[35] Gómez-Elías M.D., Rainero Cáceres T.S., Giaccagli M.M., Guazzone V.A., Dalton G.N., De Siervi A., Cuasnicú P.S., Cohen D.J., Da Ros V.G. (2019). Association between high-fat diet feeding and male fertility in high reproductive performance mice. Sci. Rep..

[36] Jia Y.-F., Feng Q., Ge Z.-Y., Guo Y., Zhou F., Zhang K.-S., Wang X.-W., Lu W.-H., Liang X.-W., Gu Y.-Q. (2018). Obesity impairs male fertility through long-term effects on spermatogenesis. BMC Urol..

[37] Chen Y., Zheng Y., Gao Y., Lin Z., Yang S., Wang T., Wang Q., Xie N., Hua R., Liu M. (2018). Single-cell RNA-seq uncovers dynamic processes and critical regulators in mouse spermatogenesis. Cell Res..

[38] Lehti M.S., Sironen A. (2016). Formation and function of the manchette and flagellum during spermatogenesis. Reproduction.

[39] Tsai-Morris C.H., Sheng Y., Lee E., Lei K.J., Dufau M.L. (2004). Gonadotropin-regulated testicular RNA helicase (GRTH/Ddx25) is essential for spermatid development and completion of spermatogenesis. Proc. Natl. Acad. Sci. USA.

[40] Dufau M.L., Tsai-Morris C.H. (2007). Gonadotropin-regulated testicular helicase (GRTH/DDX25): An essential regulator of spermatogenesis. Trends Endocrinol. Metab. TEM.

[41] Rocak S., Linder P. (2004). DEAD-box proteins: The driving forces behind RNA metabolism. Nat. Rev. Mol. Cell. Biol..

[42] Fuller-Pace F.V. (2006). DExD/H box RNA helicases: Multifunctional proteins with important roles in transcriptional regulation. Nucleic Acids Res..

[43] Schmidt E.E., Hanson E.S., Capecchi M.R. (1999). Sequence-independent assembly of spermatid mRNAs into messenger ribonucleoprotein particles. Mol. Cell. Biol..

[44] Sheng Y., Tsai-Morris C.H., Gutti R., Maeda Y., Dufau M.L. (2006). Gonadotropin-regulated testicular RNA helicase (GRTH/Ddx25) is a transport protein involved in gene-specific mRNA export and protein translation during spermatogenesis. J. Biol. Chem..

[45] Ishizawa J., Kojima K., Hail N., Tabe Y., Andreeff M. (2015). Expression, function, and targeting of the nuclear exporter chromosome region maintenance 1 (CRM1) protein. Pharmacol. Ther..

[46] Thomas J.O. (2001). HMG1 and 2: Architectural DNA-binding proteins. Biochem. Soc. Trans..

[47] Xu M., McCarrey J.R., Hecht N.B. (2008). A cytoplasmic variant of the KH-type splicing regulatory protein serves as a decay-promoting factor for phosphoglycerate kinase 2 mRNA in murine male germ cells. Nucleic Acids Res..

[48] Yoshioka H., Geyer C.B., Hornecker J.L., Patel K.T., McCarrey J.R. (2007). In vivo analysis of developmentally and evolutionarily dynamic protein-DNA interactions regulating transcription of the Pgk2 gene during mammalian spermatogenesis. Mol. Cell. Biol..

[49] Ojaghi M., Kastelic J., Thundathil J. (2017). Testis-specific isoform of angiotensin-converting enzyme (tACE) is involved in the regulation of bovine sperm capacitation. Mol. Reprod. Dev..

[50] Odroniec A., Olszewska M., Kurpisz M. (2023). Epigenetic markers in the embryonal germ cell development and spermatogenesis. Basic Clin. Androl..

[51] Cannarella R., Condorelli R.A., Mongioì L.M., La Vignera S., Calogero A.E. (2020). Molecular Biology of Spermatogenesis: Novel Targets of Apparently Idiopathic Male Infertility. Int. J. Mol. Sci..

[52] Jenkins T.G., Carrell D.T. (2011). The paternal epigenome and embryogenesis: Poising mechanisms for development. Asian J. Androl..

[53] Gaucher J., Reynoird N., Montellier E., Boussouar F., Rousseaux S., Khochbin S. (2010). From meiosis to postmeiotic events: The secrets of histone disappearance. FEBS J..

[54] Sonnack V., Failing K., Bergmann M., Steger K. (2002). Expression of hyperacetylated histone H4 during normal and impaired human spermatogenesis. Andrologia.

[55] Francis S., Yelumalai S., Jones C., Coward K. (2014). Aberrant protamine content in sperm and consequential implications for infertility treatment. Hum. Fertil..

[56] Ren S., Chen X., Tian X., Yang D., Dong Y., Chen F., Fang X. (2021). The expression, function, and utilization of Protamine1: A literature review. Transl. Cancer Res..

[57] Zhang Q., Ji S.-Y., Busayavalasa K., Shao J., Yu C. (2019). Meiosis I progression in spermatogenesis requires a type of testis-specific 20S core proteasome. Nat. Commun..

